# Kawasaki Disease in Children Older Than 10 Years: A Clinical Experience From Northwest India

**DOI:** 10.3389/fped.2020.00024

**Published:** 2020-02-14

**Authors:** Ankur Kumar Jindal, Rakesh Kumar Pilania, Sandesh Guleria, Pandiarajan Vignesh, Deepti Suri, Anju Gupta, Manphool Singhal, Amit Rawat, Surjit Singh

**Affiliations:** ^1^Allergy Immunology Unit, Department of Pediatrics, Advanced Pediatrics Centre, Postgraduate Institute of Medical Education and Research (PGIMER), Chandigarh, India; ^2^Department of Radiodiagnosis and Imaging, Postgraduate Institute of Medical Education and Research (PGIMER), Chandigarh, India

**Keywords:** adolescents, coronary artery abnormalities, intravenous immunoglobulin, Kawasaki disease, older children

## Abstract

**Background:** Kawasaki disease (KD) is predominantly seen in young children (<5 years). Diagnosis of KD is often delayed in older children and adolescents, leading to a higher risk of coronary artery abnormalities (CAAs). There is a paucity of literature on KD in older children.

**Methods:** Data were collated from a review of records of patients diagnosed with KD who were aged ≥10 years at the time of diagnosis, during the period from January 1994 to June 2019.

**Results:** Eight hundred and sixty five patients were diagnosed with KD during this period. Of these, 46 (5.3%; 26 boys and 20 girls) were aged 10 years or older at the time of diagnosis. The median age at diagnosis was 11 years (range of 10–30 years). The median interval between the of fever and the diagnosis of KD was 12 days (range of 4–30 days). Eight patients (17.4%) presented with hypotensive shock. Coronary artery abnormalities (CAAs) were seen in six patients (13.04%), and three patients had myocarditis. Patients with CAAs were found to have significantly higher median platelet counts and higher median C-reactive protein levels. First-line treatment included intravenous immunoglobulin. Adjunctive therapy was given in five patients (infliximab in four patients and steroids in one patient). The median time between the onset of fever and the administration of IVIg was 13.5 days (range of 6–2). The total duration of follow up is 2,014.5 patient-months.

**Conclusion:** Diagnosis of KD in children older than 10 years is usually delayed, and these patients are thus at a higher risk of CAAs.

## Introduction

Kawasaki disease (KD), a medium vessel vasculitis, is commonly seen in children below the age of 5. Cardiac involvement in the form of coronary artery abnormalities (CAAs) is the most significant long-term complication of KD (1, 2). In developed countries, KD is the most common cause of acquired heart disease in children. Even in developing countries, KD is now being increasingly reported, and it is emerging as one of the leading causes of acquired heart disease in children (3–5). However, no age group seems to be exempt from developing KD, and this disease can at times affect adolescents and adults as well. Diagnosis of KD often gets missed in these age groups, as many physicians may not consider KD an upfront clinical possibility in a febrile patient. This often leads to delays in diagnosis and initiation of therapy, thereby increasing the risk of developing CAAs (6).

Manlhiot et al. reported a higher incidence of CAAs in adolescents with KD, as compared to children ages 1–9 years (6). A similar study published in Indonesia found that adolescents with KD often had incomplete forms of the disease and had a higher risk of CAAs (7). A recent Japanese survey of KD, administered nationwide, has shown that 1.2% of all patients with KD were aged more than 10 years (8).

There is a paucity of literature on the clinical profile and cardiac complications of KD in adolescents and adults, especially from developing countries. We herein report our experience of KD in older patients over the last 25 years.

## Patients and Methods

Data were collated from a review of records for patients diagnosed with KD who were aged >10 years at the time of diagnosis during the period of January 1994 to June 2019 in the Pediatric Allergy Immunology Unit, Advanced Pediatrics Centre, Postgraduate Institute of Medical Education and Research (PGIMER), Chandigarh, India. Our center is a tertiary care, not-for-profit, federally funded institute in Northwest India.

Case files of patients were retrieved and clinical details were recorded on a predesigned proforma. The study was approved by the Departmental Review Board and Institute Ethics Committee. The diagnosis of KD was based on guidelines given by the American Heart Association (AHA) (1, 9). Laboratory investigations that were carried out in these patients included acute phase reactants (complete blood count, erythrocyte sedimentation rate [ESR], C-reactive protein [CRP]), urine examination, biochemistry work-up (blood urea, serum creatinine, and liver function tests), chest radiograph, and an electrocardiogram (depending on clinical requirements). 2D-echocardiography was usually performed at admission, and then again on follow up after 4–8 weeks. CAAs were initially classified based on absolute coronary artery dimensions. Since 2015, we have been using body-surface-area-based “Z” scores for the purpose of classifying the severity of CAAs. Use of 128-slice dual source computed tomography coronary angiography (CTCA) was initiated in 2014, and this imaging modality has been performed in select patients who had large or unusual CAAs, or where the visualization of coronaries was difficult because of a thick chest wall (10). Assay of serum N-terminal pro-brain natriuretic peptide (NT-proBNP) levels was initiated in our laboratory in 2014 (11).

Patients were managed using standard treatment guidelines as given by the AHA (1, 9). Intravenous immunoglobulin (IVIg−2 g/kg) given over 12–24 h was used as first-line therapy, along with oral aspirin [initially in anti-inflammatory doses (30–50 mg/kg/day) followed by antiplatelet doses (3–5 mg/kg/day)]. IVIg was not given to patients who were afebrile at presentation and who had presented late after acute stage of illness when inflammatory parameters had normalized. Adjunctive therapy (infliximab or corticosteroids) was used in selected patients with IVIg resistance or significant myocardial dysfunction.

## Results

### Clinical Profile

**Figure 1:**
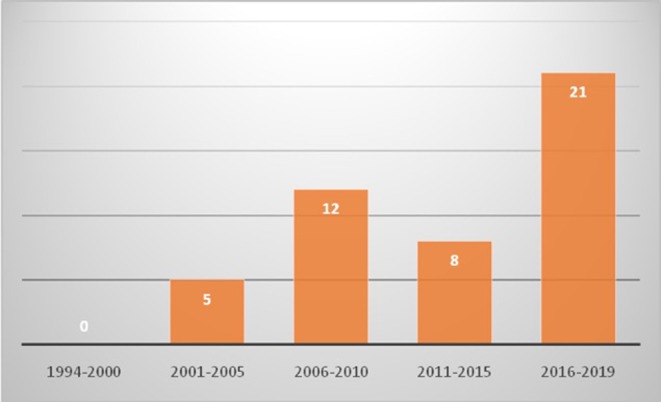
Trends of diagnosis of Kawasaki disease in children aged 10 years or older in Chandigarh, India (1994–2019) [x axis shows years and y axis shows number of cases].

Eight hundred and sixty five patients were diagnosed to have KD during this period. Of these, 46 (5.3%; 26 boys and 20 girls) were aged 10 years or more at time of diagnosis (Table 1, Figure 1). The median age at diagnosis was 11 years (range 10–30 years). All patients had a fever at presentation, and the median duration of a fever was 10 days (range 2–30 days). The median interval between the onset of fever and diagnosis of KD was 12 days (range 4–30). Eight patients (17.4%) presented with hypotensive shock. Pulmonary presentation was seen in 4 out of 46 (8.7%) patients in this group. All four patients with pulmonary presentation had pneumonia with synpneumonic effusion. Nine children (19.6%) developed arthritis. Infection-triggered KD was seen in 10 (21.7%) patients—*Staphylococcus aureus* in three patients, *Streptococcus pneumoniae* in one patient, *Mycobacterium tuberculosis* in one patient, *Pseudomonas aeruginosa* in one patient, Mycoplasma in one patient, *Burkholderia cepacia* in one patient, and *Klebsiella pneumoniae* in one patient. One patient had pyogenic skin and soft infection, but no microorganism could be isolated. BCG scar reactivation was observed in one patient.

**Table 1:** Clinical features of children with Kawasaki disease (KD) aged ≥10 years at the time of diagnosis.

**Patient characteristics**	**Results**
Median age at diagnosis (range), years	11 (10-30)
Sex	Male: 26; Female: 20
Fever	46/46 (100%)
Median duration of fever	10 (2-30)
Day of diagnosis (days)	12 (4-30)
Rash	34/46 (73.9%)
Oral cavity and mucosal changes	36/46 (78.3%)
Conjunctival redness	29/46 (63%)
Cervical lymphadenopathy	28/46 (60.9%)
Dorsal edema	11/46 (23.9%)
Periungual desquamation	45/46 (97.8%)
Day of desquamation (days)	10 (5-20)
Irritability	3/46 (6.5%)
Arthritis	9/46 (19.6%)
Hypotensive shock	8/46(17.4%)
Sterile pyuria	6/46 (13%)
Pulmonary manifestations	4/46 (8.7%)
Significant myocardial dysfunction	3/46 (6.5%)
Infection-triggered KD	10/46 (21.7%)

### Laboratory Characteristics

Laboratory investigations of patients at the time of diagnosis are shown in Table 2. The mean hemoglobin concentration was 106.7 ± 18.3 g/L. The median value of maximum platelet count was 541 × 10^9^/L. Five patients had thrombocytopenia at time of presentation, and of these, two patients had CAAs and one patient had low ejection fraction. Median ESR and CRP were 42.5 mm in the first hour and 43 mg/L, respectively. Aspartate (AST) and alanine transaminase (ALT) values were available in 41 and 39 patients, respectively. AST and ALT values were elevated in 18 out of 41 patients (43.9%) and 18 out of 39 (46.2%) patients, respectively. Most patients had mild elevation in transaminases (i.e., <2 times). Six patients had significant elevations in transaminases, and of these, one patient had levels more than 10 times the normal level. Transaminase levels normalized after treatment. We were able to monitor serum procalcitonin in 14 patients, and median levels were 1.18 ng/mL (range 0.05–33) (Normal <0.5). Nine out of fourteen patients (64.3%) had elevated procalcitonin value, and of these, four had infection-triggered KD. In 21 patients, we were able to perform NT-pro-BNP, and the median value was 225 pg/mL (range 29–9,435) (Normal < 125). NT-pro-BNP values were found to be elevated in 16 out of 21 (76.2%) patients.

**Table 2:** Laboratory investigations.

**Characteristics**	**Results**
Hemoglobin (g/L); Mean ± SD	106.7 ± 18.3
White blood cell counts (×10^9^/L); Median (range)	13.45 (2.20–38.65)
Platelet counts at admission (×10^9^/L); Median (range)	333 (21.5, 1121)
Maximum platelet counts (×10^9^/L); Median (range)	541 (183, 1470)
Erythrocyte sedimentation rate (mm/hour); Median (range)	42.5 (2, 118)
C-reactive protein (<6 mg/L); Median (range)	43 (1.24, 269)
Procalcitonin (<0.50 ng/mL); Median (range)	1.18 (0.05, 33)
Aspartate transaminase (<40 U/L); Median (range)	37 (9, 585)
Alanine transaminase (<41 U/L); Median (range)	36.5 (7, 866)
Serum albumin (mg/dL); Mean ± SD	3.07 ± 0.70
Sterile pyuria, *n* (%)	6/42 (14.3%)

### Cardiac Complications

CAAs were seen in six (13.04%) patients: two had isolated left main coronary artery (LMCA) aneurysms; one had isolated left anterior descending artery (LAD) aneurysms; one had LAD and right coronary artery (RCA) aneurysms; one had aneurysms in LMCA, LAD, and RCA; and one had aneurysms in all four coronary arteries. Frequency of involvement of coronary arteries was as follows: LMCA−4 out of 12 (33.3%); LAD−4 out of 12 (33.3%); RCA−3 out of 12 (25%); and left circumflex coronary artery (LCx)−1 out of 12 (8.3%). Giant aneurysms were seen in two patients—one had involvement of the LAD and RCA, while the other had involvement of the LAD, RCA, and LCx. Three patients had severe myocardial dysfunction secondary to myocarditis. One patient had a stormy course of illness and developed ventricular premature complexes, severe myocarditis, pericardial effusion with tamponade; this patient required a pleuro-pericardial window.

### Treatment

Median time between onset of fever and administration of IVIg was 13.5 days (range 6–32 days). Seven patients were not offered treatment, as they had presented late in convalescent phase when the fever had subsided, inflammatory markers had settled, and 2D-echocardiography was normal. Adjunctive therapy was required in five patients (repeat IVIg and infliximab in one; infliximab alone in three; and methylprednisolone in one) (Table 3). Total duration of follow up was 2,014.5 patient-months. Five patients were not on regular follow up at time of this analysis. However, none of them had developed CAAs during the acute phase. No mortality was seen in this cohort, and none of the patients with CAAs developed thrombosis or acute coronary artery events on follow up.

**Table 3:** Treatment details.

**Characteristics**		***n* = 46 (%)**
Treatment with IVIg		39/46 (84.7%)
Adjunctive treatment		5/46 (10.9%)
(i)	Infliximab	3 (6.5%)
(ii)	Second dose of IVIg and infliximab	1 (2.2%)
(iii)	Methylprednisolone pulse followed by tapering doses of prednisolone	1 (2.2%)

*IVIg, intravenous immunoglobulin*.

We compared the clinical profile of patients with and without CAAs (Table 4). Patients with CAAs were found to have significantly higher median maximum platelet count and higher median CRP. Pulmonary presentation was significantly more common in patients with CAAs, and these patients required adjunctive therapy more commonly. Although the median time between onset of fever and diagnosis of KD was relatively higher in patients with CAAs as compared to those without CAAs (14.5 days vs. 12 days), the difference was not statistically significant. Thrombocytopenia during the acute stage of KD was also found to be more common in patients with CAAs. All other clinical features and laboratory findings were similar in the two groups.

**Table 4:** Comparison of clinical and laboratory features of patients of Kawasaki disease (KD) with and without coronary artery abnormalities (CAAs).

**Characteristics**	**KD without CAAs (*n* = 40)**	**KD with CAAs (*n* = 6)**	***p*-value**
Interval between onset of fever and diagnosis (days), Median (IQR)	12 (10,20)	14.5 (6, 15.5)	0.53
Hypotensive shock, *n* (%)	7/40 (17.5%)	1/6 (16.7%)	0.96
Arthritis, *n* (%)	8/40 (20%)	0	0.23
Infections, *n* (%)	9/40 (22.5%)	2/6 (33.3%)	0.56
Pulmonary presentation, *n* (%)	2/40 (5%)	3/6 (50%)	**0.001**
Diarrhea, *n* (%)	8/40 (20%)	1/6 (16.7%)	0.82
Hemoglobin (g/L), Mean ± SD	102.5 ± 13.5	107.4 ± 18.9	0.458
Total leucocyte count (×10^9^/L), Median (IQR)	12.7 (8.67, 18.4)	15.9 (13.7, 23.8)	0.09
Platelet counts at admission (×10^9^/L), Median (IQR)	333.0 (233.0, 464.7)	359.5 (103.7, 931.3)	1.00
Thrombocytopenia at admission (<150 × 10^9^/L), *n* (%)	3/40 (7.5%)	2/6 (33.3%)	0.06
Maximum recorded platelet count (×10^9^/L), Median (IQR)	502.0 (409.0, 649.0)	817.5 (635.2, 994.5)	**0.04**
Erythrocyte sedimentation rate (mm/hour), Median (IQR)	36 (20.5, 60.0)	59 (44.3, 77.3)	0.18
C-reactive protein (mg/L), Median (IQR)	40.0 (6.59, 64.5)	115.0 (48.5, 249.0)	**0.05**
Albumin (g/L), Mean ± SD	2.81 ± 0.75	3.12 ± 0.70	0.337
Aspartate transaminase (U/L), Median (IQR)	34 (24.0, 51.0)	76.0 (32.3, 82.3)	0.13
Alanine transaminase (U/L), Median (IQR)	35 (29.5, 64.7)	51.0 (26.3, 67.0)	0.66
NT-Pro BNP (pg/mL), Median (IQR)	225.0 (122.2, 600.0)	231.5 (116.3, 447.3)	0.788
Adjunctive therapy, *n* (%)	2/40 (5%)	3/6 (50%)	**0.001**
Day of administration of IVIg, Median (IQR)	13.5 (10.8, 21.3)	15.0 (10.3, 17.0)	0.743

*CAAs, coronary artery abnormalities; IQR, interquartile range; IVIg, intravenous immunoglobulin; pg, pictogram; KD, Kawasaki disease; NT-Pro BNP, N-terminal pro-brain natriuretic peptide; SD, standard deviation. Bold values indicate p value < 0.05*.

## Discussion

KD is the most common childhood vasculitis, and it usually affects children below the age of 5. Diagnosis of KD at extremes of age can, however, pose several diagnostic and therapeutic problems. We, along with others, have previously reported our experience of KD in infants below 6 months of age (12, 13). These studies have shown that infants with KD are at a high risk of developing CAAs despite timely diagnosis and treatment. Incomplete forms of KD are also very common in this age group (12, 13).

Diagnosis of KD in older children and adolescents is often challenging, as the treating physician may not consider this condition during their differential diagnosis of a febrile child. There is a paucity of literature on the clinical presentation of KD in older children and adolescents from developing countries. Manlhiot et al. have recently published their experience on KD occurring in extremes of age and have shown that 6% of patients in their cohort were aged 9 years or older—making their findings similar to ours (6). However, a recent nationwide Japanese survey has shown that only 1% of patients with KD were aged more than 10 years at the time of diagnosis (8). Similarly, a study by Advani et al. has found that 17 out of 1,150 (1.5%) of such cases involved patients aged more than 10 years (7). The percentage of cases of KD in older age groups varies from 1 to 7% in different studies (6, 7, 14–17), but Cai et al. have reported a figure as high as 17%. However, they have included children above the age of 5 in their analysis (17).

We have a cohort of 865 patients that were diagnosed with KD from the period of January 1994 to June 2019. Forty-six (5.3%) amongst these were aged 10 years or older at the time of diagnosis. One possible explanation for the relatively higher proportion of older patients with KD in our cohort is that we may be missing KD in infants and young children, in whom this condition often gets confused with febrile exanthemata (18, 19).

The clinical profile of patients with KD in this cohort has been compared with previously published studies (Table 5). Amongst the principal clinical features, periungual desquamation was found in 98% of cases, followed by oral cavity and lip changes (78%), skin rash (74%), conjunctival injection (63%), lymphadenopathy (61%), and dorsal edema (24%). However, as several patients had presented late, it is possible that some of the clinical features may have been missed altogether.

**Table 5:** A review of all reported studies on Kawasaki disease in older age groups.

	**Momenah et al. Canada (16)**	**Stockheim et al. USA (15)**	**Cai et al. China (17)**	**Nagamori et al. Japan (14)**	**Manlihot et al. USA (6)**	**Advani et al. Indonesia (7)**	**Present study**
Total no. of patients	133	500	113	650	1,374	1,150	865
No. of patients in older age group (%)	10 (7.5)	28 (5.6)	20 (17.7)	14 (2.2)	87 (6)	17 (1.5)	46 (5.3)
Age group (years)	≥9	≥8	≥5	>7	>9	>10	≥10
Median age in years (range)	NR	9 (8-15)	NR	8	NR	11.2 (10-16)	11 (10-30)
Mean age in years	10.9 ± 0.46	NR	8.22 ± 3	NR	NR	11.25 ± 1.2	16.3 ± 4.1
Male: female	1:1	2.5:1	2.33:1	NR	2:1	4.6:1	1.3:1
Fever duration (median, days)	10.8	10.5	10.8	5 (3-12)	9 (5-42)	NR	10 (2-30)
Rash (%)	NR	25 (89)	14 (70)	NR	69 (83)	10 (59)	34 (73.9)
Conjuctival injection (%)	NR	28 (100)	17 (85)	NR	70 (84)	11 (65)	29 (63)
Oral mucosal changes (%)	NR	27(96)	18 (90)	NR	73 (88)	13(77)	36 (78.3)
Cervical adenopathy (%)	NR	17 (61)	17 (85)	5 (36)	49 (59)	14 (82)	28 (60.9)
Extremity changes (%)	NR	28 (100)	17 (85)	9 (64)	63 (76)	7 (41)	45(97.8)
Hemodynamic instability/KDSS	NR	NR	NR	NR	NR	NR	8 (17.4)
Incomplete KD (%)	NR	1 (3.57)	5 (25)	4 (29)	36	10 (59)	20 (43)
IVIG used (%)	10 (100)	22 (78.6)	19 (95)	14 (100)	68 (81)	NR	39 (84.8)
IVIG resistance (%)	2 (20)	–	6 (30)	1 (7.14)	14 (17)	NR	none
Coronary abnormalities (%)	8 (40)	6 (21)	12 (60)	2 (14)	22 (25)	8 (47)	6 (13.04)

*IVIG, intravenous immunoglobulin; KDSS, Kawasaki Disease Shock Syndrome; NR, not reported*.

Diagnosis of KD should ideally be made before day 10 of illness, so that timely treatment can be instituted to prevent the development of CAAs (1, 20). In our cohort, the median day of diagnosis was 12 days. Manhliot et al. have also shown that the diagnosis of KD in children older than 9 years was delayed until day 12 (6). Seven patients in our cohort (15.2%) were not offered treatment, as they had presented late and their fever had already subsided. Similarly, 19% of the patients were not treated in the study published by Manlihot et al. (6), and 22% of the patients in the study published by Stockheim et al. (15).

The most significant morbidity in patients with KD is due to development of CAAs. Approximately 25% of KD cases develop CAAs if the disease remains untreated. With timely initiation of IVIg, <5% patients will go on to develop CAAs (1). Risk of CAAs is higher in older children with KD as compared to their younger counterparts (6, 15–17). Delay in diagnosis and initiation of treatment is considered to be the most important risk factor for development of CAAs in these patients (6). In the present cohort, 13% of cases developed CAAs. Our results are in consonance with the findings of previous studies (6, 7, 15–17).

It has also been observed by several authors that incomplete presentations are more common in older patients with KD (up to 59% in various series) (6, 7, 14, 17). We observed incomplete KD in 43% of our patients. Delay in diagnosis of KD has been found to be the most important risk factor for the development of CAAs in this age group. Manlihot et al. reported that unlike young infants, these patients have not been found to have other risk factors contributing to the development of CAAs, such as low serum albumin, hemoglobin, and platelet counts. The mean hemoglobin levels seen in the study by Manlihot et al. were 123 ± 15 g/L (6), while the mean levels in our cohort were 106.7 ± 18.3 g/L. Mean albumin concentration seen in our series was 31 ± 7 g/L. This was also lower than mean serum albumin level as reported by Manlihot et al. (38 ± 8 g/L) (6). It is likely that the underlying nutritional status of our cohort was responsible for these apparent differences. Mean hemoglobin and mean albumin levels were not found to be different in children with or without CAAs in our study. Median platelet counts in the study by Manlihot et al. were reported to be 268 × 10^9^/L (6), while the median platelet count at presentation in our cohort was 333 × 10^9^/L. We also observed that the median CRP was significantly higher in our patients who developed CAAs, as compared to our patients who did not develop CAAs.

In our cohort, the children who developed CAAs had a relatively higher median interval between the onset of fever and the diagnosis of KD when compared to children who did not develop CAAs. Similarly, pulmonary presentation was more common in patients with CAAs. We have previously reported that delays in diagnosis and initiation of treatment are more common in patients with KD who also have a pulmonary presentation (21). Although this increased risk of CAAs in KD in older age groups can be ascribed to delays in diagnosis and treatment, one cannot be categorical (as there may be several confounding factors at play). Further, it would be imprudent to draw conclusions when the numbers are small.

Eladawy et al. reported that 48.5% of patients with KD had one or more liver enzyme abnormalities. The authors have also shown that most liver enzyme elevations were subclinical and <2 times above the reference values. Elevation of liver enzymes were found to be associated with increased risk of IVIg resistance (22). In our study, elevated liver transaminases were seen in 46% of patients, and there was no difference in the frequency of liver enzyme abnormalities in patients with and without CAAs (Table 4).

Approximately 5% of children with KD can also present with hemodynamic instability and hypotensive shock—termed KD Shock Syndrome (KDSS) (1, 23, 24). In our study, 8 out of the 46 cases (17.4%) had KDSS-like presentation and initially, the possibility of toxic shock syndrome was considered in all of them. There was a significant delay in the diagnosis of KD in these patients—in one case the diagnosis was delayed until 3 weeks while in two cases the diagnosis cold be made only after 4 weeks. CAAs were seen in one patient with KDSS. The relatively higher proportion of patients with KDSS in our cohort could be a reflection of late diagnosis as well as delays in the initiation of therapy.

While managing patients with KD in older age groups, it needs to be kept in mind that getting an appropriate acoustic window for coronary artery evaluation may be difficult. In such situations, it may be difficult to rely completely on 2D-echocardiography findings, and one may have to pre-emptively carry out CTCA in selected patients (25). We carried out this investigation in 8 out of 46 patients.

In conclusion, our study shows that 5.3% of patients with KD were aged 10 years or older at time of diagnosis. Older children with KD appear to have significant delays in diagnosis of the disease and a higher chance of development of CAAs. The strength of this study is that all patients have been diagnosed and treated on the basis of uniform protocols and by a team with more than 25 years of experience of managing this condition. The obvious lacuna is the small cohort size and the fact that it is a single center retrospective study. Multicenter, large-sample, prospective studies would help in further understanding the significance of our findings.

## Data Availability Statement

The datasets generated for this study are available on request to the corresponding author.

## Ethics Statement

The study protocol was approved by the Departmental Review Board and Institutional Ethics Committee of the Postgraduate Institute of Medical Education and Research, Chandigarh, India. Diagnosis of KD was based on guidelines given by American Heart Association (AHA) (1, 9). As this manuscript pertains to collation of anonymized retrospective data from clinic records, acquiring consent was not necessary.

## Author Contributions

AJ and RP: conceptualization of the study, data collection, patient management, writing of the initial draft, editing of the manuscript, and review of the literature. SG, PV, DS, AG, and AR: patient management and review of literature, revision of manuscript. MS: radiological investigations, patient management, and review of literature. SS: conceptualization of the study, data collection, patient management, critical revision of manuscript, review of literature, and final approval of the manuscript.

### Conflict of Interest

The authors declare that the research was conducted in the absence of any commercial or financial relationships that could be construed as a potential conflict of interest.
